# Network Toxicology and Transcriptomic Analyses Reveal Ferroptosis-Related Neurotoxicity of Rotenone as an Environmental Hazardous Compound

**DOI:** 10.3390/cells15110959

**Published:** 2026-05-22

**Authors:** Yimeng Chen, Ding Zhang, Jiajia Ma, Huixin Li, Jingrong Xu, Cuixia Ma, Yuqian Liu, Zhenbing Zhao, Garry P. Duffy, Jun Ma, Huixian Cui

**Affiliations:** 1 Hebei Medical University-University of Galway Stem Cell Research Center, Hebei Medical University, Shijiazhuang 050017, China; 2 Hebei Research Center for Stem Cell Medical Translational Engineering, Shijiazhuang 050017, China; 3 Hebei Technology Innovation Center for Stem Cell and Regenerative Medicine, Shijiazhuang 050017, China; 4 Hebei International Joint Research Center for Stem Cell and Regenerative Medicine, Shijiazhuang 050017, China; 5 Department of Electronic and Communication Engineering, North China Electric Power University, Baoding 071003, China; 6 Anatomy and Regenerative Medicine Institute, College of Medicine Nursing and Health Sciences, University of Galway, H91 W2TY Galway, Ireland; 7 Human Anatomy Department, Hebei Medical University, Shijiazhuang 050017, China

**Keywords:** rotenone, ferroptosis, Parkinson’s disease, network toxicology, transcriptomics, lipid peroxidation, neurotoxicity

## Abstract

**Highlights:**

**What are the main findings?**

- Network toxicology and Parkinson’s disease transcriptomic analyses identify an 11-gene, PD-contextualized, ferroptosis-associated response module linked to rotenone-induced neuronal injury.
- Rotenone induces ferroptosis-associated neurotoxicity in SH-SY5Y cells, as supported by ultrastructural alterations, lipid peroxidation, and GPX4–ACSL4 dysregulation.

**What are the implications of the main findings?**

- These findings provide a disease-informed framework linking environmental rotenone exposure to ferroptosis-associated neuronal injury in a Parkinson’s disease-relevant context.
- The identified Fer-1-responsive gene module provides testable candidates for future mechanistic studies of ferroptosis-associated neurodegeneration.

**Abstract:**

**Background:** Rotenone is a widely used environmental pesticide, and epidemiological studies suggest that exposure is associated with an increased risk of Parkinson’s disease (PD); however, the molecular toxicological basis of this association remains incompletely defined. Ferroptosis is an iron-dependent, lipid peroxidation-driven form of regulated cell death that is relevant to PD and other neurodegenerative disorders. In this study, we provide disease-contextual functional evidence linking ferroptosis to rotenone-induced PD-like neurotoxicity. **Methods:** We combined network toxicology, human PD substantia nigra transcriptomic analysis using GSE7621, and SH-SY5Y cell-based validation. Rotenone-associated targets were predicted and analyzed for ferroptosis-related enrichment, PD transcriptomic signatures were used for disease-contextual candidate prioritization, and selected findings were validated using qPCR, CCK-8, Western blotting, C11-BODIPY lipid peroxidation staining, and transmission electron microscopy. **Results**: By further integrating a human PD substantia nigra transcriptomic dataset (GSE7621), we prioritized an 11-gene, PD-contextualized ferroptosis-associated candidate module (*LIPF*, *FAM170A*, *MCHR1*, *IL17A*, *MYB*, *GFAP*, *ARMC3*, *GKN1*, *GATA3*, *IL17F*, and *TEKT1*). In SH-SY5Y cells, rotenone exposure consistently upregulated this candidate transcriptional module, and this induction was broadly attenuated by the ferroptosis inhibitor ferrostatin-1 (Fer-1). In parallel, orthogonal functional assays supported an iron- and lipid peroxidation-driven injury state under rotenone exposure that was suppressible by ferroptosis inhibition and iron chelation. Finally, we further performed an exploratory drug–gene association screen to prioritize clinically available candidates, and a limited qPCR check suggested that several selected compounds partially attenuated representative hub-gene induction under rotenone exposure. **Conclusions**: Collectively, these findings provide disease-contextual and experimentally supported evidence linking rotenone exposure to ferroptosis-associated neurotoxicity, and identify a ferroptosis-responsive transcriptional module for future hypothesis-driven mechanistic investigation.

## 1. Introduction

Rotenone, a naturally occurring plant-derived compound, has long been used as a botanical insecticide and fish poison in agriculture and domestic settings [1]. Its strong lipophilicity and persistence enable its accumulation in the environment and biological systems, raising considerable concern regarding its neurotoxic potential [2]. Environmental monitoring studies have detected Rotenone residues in surface water, sediment, and agricultural products, with reported concentrations ranging from nanomolar to low micromolar levels (e.g., 0.1–10 μg/L), depending on local usage and degradation rates [3]. Chronic low-dose exposure through drinking water or food consumption raises additional concerns regarding its long-term neurotoxic effects [4,5]. Numerous animal and cellular studies have demonstrated that rotenone exposure disrupts mitochondrial function by inhibiting complex I of the respiratory chain, leading to dopaminergic neuronal degeneration and Parkinson’s disease (PD)-like motor symptoms [6]. Owing to these characteristics, rotenone is widely used as a chemical model to study environmentally induced parkinsonism [7,8].

Recent studies have implicated ferroptosis, a regulated form of cell death characterized by iron accumulation and lipid peroxidation, in rotenone-induced neuronal injury. Here, we provide additional disease-contextual evidence and mechanistic validation to consolidate ferroptosis as a PD-contextualized rotenone-responsive injury program [9]. Studies have shown that rotenone promotes intracellular iron overload, suppresses glutathione metabolism, and induces oxidative lipid damage—hallmarks of ferroptotic pathways [10,11,12]. However, the molecular regulatory networks linking rotenone exposure to ferroptosis remain largely undefined. Moreover, while rotenone is extensively used to mimic PD pathology in experimental models, few studies have utilized human-derived transcriptomic datasets to explore whether rotenone-related toxic signatures are reflected in real PD patient tissues.

PD is increasingly recognized as the product of complex interactions between genetic susceptibility and environmental exposures [13]. Integrating rotenone-based neurotoxic mechanisms with PD-derived molecular data may therefore provide meaningful insights into the role of ferroptosis in environmentally triggered neurodegeneration.

Traditional toxicological approaches often examine a single target or pathway in isolation, which may fail to capture the systemic and multi-target nature of environmental toxicants like rotenone. To address these gaps, we employed a network toxicology-based transcriptomic approach to dissect the potential neurotoxic mechanisms of rotenone. Specifically, we utilized the GSE7621 dataset derived from the substantia nigra of PD patients as a clinical context benchmark for rotenone-induced toxicity. This approach is justified by rotenone’s recognized ability to reproduce key molecular and phenotypic features of PD in animal and in vitro models. Furthermore, we integrated compound–target prediction using SwissTargetPrediction and CTDs, constructed a compound–target–pathway network, and highlighted ferroptosis-related regulatory axes. Subsequent functional verification was performed in rotenone-treated neuronal cells to validate the expression patterns of key targets.

By integrating public transcriptomic data with predictive toxicology modeling, this study aimed to reveal a ferroptosis-centered regulatory framework underpinning rotenone-induced neurotoxicity. The findings contribute to a more comprehensive understanding of rotenone’s toxic mechanisms as an environmental hazard and offer molecular clues for risk evaluation and preventive strategies.

## 2. Materials and Methods

### 2.1. Network Toxicology Analysis

To explore the potential toxicological mechanisms of rotenone, we performed a network toxicology analysis based on compound–target–pathway relationships. First, the chemical structure of rotenone was retrieved in SMILES format from the PubChem database (CID: 6758; https://pubchem.ncbi.nlm.nih.gov/compound/6758, accessed on 18 January 2026) and used as input into the SwissTargetPrediction platform (http://www.swisstargetprediction.ch/, accessed on 18 January 2026) to predict probable human protein targets. The organism was set to *Homo sapiens*, and targets with a probability score > 0 were retained.

In parallel, we queried the Comparative Toxicogenomics Database (CTD) (https://ctdbase.org/, accessed on 18 January 2026) using “rotenone” as a chemical keyword to extract known human gene interactions and pathway associations based on curated experimental evidence. Only interactions supported by direct evidence or inferred from human data were included.

All identified targets from SwissTargetPrediction and CTD were combined, duplicates removed, and the final list was used for downstream analysis. GO and KEGG enrichment analyses were conducted using the clusterProfiler R package (version 4.8.3) in R software (version 4.3.1) to identify overrepresented biological processes and pathways. Enrichment results were further filtered to highlight ferroptosis-related pathways, including terms containing “ferroptosis”, “iron”, “lipid peroxidation”, or “oxidative stress”. A rotenone–target–pathway tripartite interaction network was constructed and visualized using Cytoscape (version 3.9.1).

### 2.2. Data Sources and Differential Expression Analysis

Gene expression data were retrieved from the Gene Expression Omnibus (GEO) database using the GEOquery R package (version 2.68.0) [14]. The Parkinson’s disease dataset GSE7621 [15] (substantia nigra; 25 samples: 9 controls and 16 PD patients) was analyzed. The GSE7621 dataset is a publicly available resource containing de-identified human substantia nigra transcriptomic data generated from previously published studies; therefore, no additional institutional ethical approval was required for the present secondary analysis. Expression data were normalized using the limma package (version 3.56.2) [16]. Differential expression analysis was performed between PD and control groups, with DEGs defined as |log2FC| > 1 and adjusted *p* value (Benjamini–Hochberg FDR) < 0.05. Heatmaps and volcano plots were generated using ggplot2 R package (version 3.5.1) [17]. To identify ferroptosis-related DEGs, we queried GeneCards (https://www.genecards.org/, accessed on 18 January 2026) with the term “ferroptosis” and used the resulting gene list as the reference set for DEG overlap analysis after gene symbol standardization and de-duplication. In parallel, FerrDb (http://www.zhounan.org/ferrdb/, accessed on 18 January 2026) and other curated ferroptosis resources were used at the pathway/program level to support interpretation and contextualization of ferroptosis-related processes (as described in the network toxicology analysis).

### 2.3. Cluster Analysis

Consensus clustering was performed using the ConsensusClusterPlus R package (version 1.64.0) [18]. with the partitioning around medoids (PAM) algorithm and Euclidean distance. The number of clusters (*k*) was evaluated from 2 to 9, with 80% item resampling repeated for 1000 iterations. The t-distributed Stochastic Neighbor Embedding (t-SNE) technique was applied to intuitively and effectively visualize clustering results [19]. The optimal number of clusters was determined based on the consensus cumulative distribution function (CDF) and delta area plots, together with cluster stability across resampling iterations.

### 2.4. WGCNA

WGCNA was performed using the WGCNA R package (version 1.73) in R software (version 4.3.1) to identify relevant gene modules, explore relationships between gene networks and phenotypes, and investigate hub genes within the network [20]. The optimal soft-thresholding power was calculated using the pickSoftThreshold function, which yielded a value of 7. The soft-thresholding power was selected to approximate a scale-free topology while maintaining adequate mean connectivity. A scale-free network was constructed based on this threshold to create a topological overlap matrix and hierarchical clustering. Gene modules were identified using a dynamic tree cut, with a minimum module size of 50 genes. Module eigengenes were calculated to determine inter-module correlations, and modules with a correlation greater than 0.4 were merged [21]. Finally, Spearman’s correlation analysis was used to assess the association between modules and clinical traits [22].

### 2.5. Enrichment Analysis (GO/KEGG/GSEA)

GO analysis is a commonly used method for large-scale functional enrichment studies, encompassing biological processes (BP), molecular functions (MF), and cellular components (CC). KEGG is a widely used database that provides information on genomes, biological pathways, diseases, and drugs [23]. GO and KEGG enrichment analyses were performed using the clusterProfiler R package (version 4.8.3) in R software (version 4.3.1). *p* values were adjusted using the Benjamini–Hochberg method, and terms with FDR < 0.05 were considered significant. Enrichment results were visualized using ggplot2 R package (version 3.5.1) [24].

We conducted GSEA [25] based on the gene expression profiling of PD samples from the GSE7621 dataset to explore the differences in BP between the different groups. GSEA was performed using gene sets fromthe Molecular Signatures Database (MSigDB; https://www.gsea-msigdb.org/gsea/msigdb/, accessed on 18 January 2026) (C2: curated gene sets, KEGG collection; gene symbols), and an FDR < 0.25 was considered significant. It is commonly applied to assess changes in expression data pathways and biological process activity. We used GSEA and the “c2.cp.kegg.v6.2.symbols.gmt“ gene set obtained from the MSigDB database [26]. An FDR of less than 0.25 was considered to indicate significant enrichment.

### 2.6. PPI Network Construction

The STRING database (https://string-db.org/, accessed on 18 January 2026) is a high-confidence bioinformatics resource designed for the systematic analysis and prediction of protein–protein interactions (PPIs), integrating a wide range of data sources, such as experimental findings, computational predictions, and curated biological pathways, to support the in-depth exploration of molecular interaction networks [27]. Using the STRING database, we constructed a PPI network for differentially expressed ferroptosis-related genes, with a minimum required interaction score of 0.7. Spearman correlation analysis was used to examine the relationships between key ferroptosis-related genes identified in the study [22].

### 2.7. Quantitative Real-Time PCR Analysis

We used a toxin-induced cell model to validate gene expression. Human neuroblastoma SH-SY5Y cells were purchased from the Institute of Basic Medical Sciences, Chinese Academy of Medical Sciences & Peking Union Medical College (Beijing, China). SH-SY5Y cells were treated with rotenone (400 nM; Sigma-Aldrich, St. Louis, MO, USA; Cat. No. R8875; vehicle: DMSO, Wuhan Servicebio Technology Co., Ltd., Wuhan, China; Cat. No. GC203006-10ml; final ≤0.1% v/v, matched across groups) for 24 h, and then cells were collected for further analysis. Cell lysis buffer(RLT Lysis Buffer, Qiagen, Hilden, Germany)can rapidly lyse cells, inactivate RNA enzymes, and remove genomic DNA residues using DNA removal/RNA adsorption universal column adsorption technology. At the same time, it efficiently binds to the silica membrane of the adsorption column under the condition of ethanol-assisted binding and then removes cell metabolites and proteins, such as impurities, to obtain purity after several washes and centrifugation of total RNA.

The total RNA concentration and purity were determined using a NanoDrop One spectrophotometer (Thermo Fisher Scientific, Waltham, MA, USA). Reverse transcription was performed on a PCR instrument (Bio-Rad Laboratories, Hercules, CA, USA) using GoScript™ Reverse Transcription Mix, Random Primers (Promega, Madison, WI, USA; Cat. No. A2801). Quantitative PCR was performed using GoTaq qPCR Master Mix (Promega, Madison, WI, USA; Cat. No. A6002) on a LightCycler 480 II real-time PCR system (Roche Diagnostics, Mannheim, Germany). According to our previous study, PCR conditions were 95 °C for 30 s, 95 °C for 5 s, 60 °C for 10 s, and 72 °C for 60 s over 45 cycles. Gene expression changes were determined by the 2-ΔΔCt method using β-actin as an internal reference. The specific primer sequences for the genes of interest are listed in Table 1.

### 2.8. Cell Viability Assay (CCK-8) and Dose Selection

SH-SY5Y cells were seeded into 96-well plates (5–8 × 10^3^ cells/well) and allowed to adhere overnight. For dose–response experiments, SH-SY5Y cells were exposed to increasing concentrations of rotenone (0, 100, 400, and 800 nM) for 24 h (Supplementary Figure S1). Based on these preliminary experiments, 400 nM rotenone was selected for subsequent mechanistic assays because it produced a moderate and reproducible reduction in cell viability while avoiding excessive cytotoxicity. For rescue experiments, cells were pretreated with ferrostatin-1 (Fer-1, 2 µM; Beyotime Biotechnology, Shanghai, China; Cat. No. Y240805) or the iron chelator deferoxamine (DFO, 100 µM; Beyotime Biotechnology, Shanghai, China; Cat. No. SH5266) for 1 h, followed by co-treatment with rotenone (400 nM) for 24 h. The final DMSO concentration was kept constant across all conditions (≤0.1%, *v*/*v*). Cell viability was measured using theCCK-8 assay kit (Wuhan Servicebio Technology Co., Ltd., Wuhan, China; Cat. No. G4103-1ML) according to the manufacturer’s instructions: CCK-8 reagent was added to each well and incubated for 1–2 h at 37 °C, and absorbance was read at 450 nm using a microplate reader. Viability was calculated as a percentage of the vehicle control. Each condition was tested in triplicate wells and repeated in at least three independent experiments.

### 2.9. Western Blotting

After the indicated treatments, SH-SY5Y cells were lysed in RIPA buffer (Wuhan Servicebio Technology Co., Ltd., Wuhan, China; Cat. No. G2002-30ML) supplemented with PMSF (Wuhan Servicebio Technology Co., Ltd., Wuhan, China; Cat. No. G2008-1ML), and protein concentrations were determined by a BCA protein assay kit (Wuhan Servicebio Technology Co., Ltd., Wuhan, China; Cat. No. G2026-200T). Equal amounts of protein were separated by SDS–PAGE and transferred to PVDF membranes (Merck, Darmstadt, Germany). Membranes were blocked with 5% non-fat milk (Beyotime Biotechnology, Shanghai, China; Cat. No. P0216-300g) and incubated overnight at 4 °C with primary antibodies against ACSL4 (Santa Cruz Biotechnology, Dallas, TX, USA; Cat. No. sc-271800; 1:500), GPX4 (anti-Glutathione Peroxidase 4 [EPNCIR144], Abcam, Cambridge, UK; Cat. No. ab125066; 1:1000), and GAPDH (Anti-GAPDH antibody [EPR16891]—Loading Control, Abcam, Cambridge, UK; Cat. No. ab181602; 1:10,000). After incubation with HRP-conjugated secondary antibodies, including goat anti-rabbit IgG-HRP (Abcam, Cambridge, UK; Cat. No. ab6721; 1:10,000) and goat anti-mouse IgG H&L (HRP) (Abcam, Cambridge, UK; Cat. No. ab6789; 1:10,000), signals were visualized using an enhanced chemiluminescence reagent (NcmECL High, NCM Biotech, Suzhou, China; Cat. No. P2100), and band intensities were quantified using ImageJ (version 1.54p; National Institutes of Health, Bethesda, MD, USA) and normalized to GAPDH.

### 2.10. Lipid Peroxidation Assay (BODIPY 581/591 C11)

Lipid peroxidation was assessed using the BODIPY 581/591 C11 probe (Beyotime Biotechnology, Shanghai, China; Cat. No. S0043S). After the indicated treatments (Control, ROT, ROT + Fer-1), SH-SY5Y cells were incubated with BODIPY 581/591 C11 (2 µM) at 37 °C in a humidified CO_2_ incubator for 30 min, protected from light. Cells were then gently washed with PBS and immediately imaged using a fluorescence microscope. Oxidation of the probe was reflected by a shift from red to green fluorescence, and images were acquired under identical exposure settings across groups for comparison.

### 2.11. Transmission Electron Microscopy (TEM)

After the indicated treatments (Control, ROT, ROT + Fer-1), SH-SY5Y cells were collected and fixed in 2.5% glutaraldehyde at 4 °C, followed by post-fixation in 1% osmium tetroxide. Samples were dehydrated through a graded ethanol series, embedded in epoxy resin, and sectioned into ultrathin slices (~70 nm). Sections were stained with uranyl acetate and lead citrate and examined using an HT7700 transmission electron microscope (Hitachi, Tokyo, Japan). Multiple randomly selected fields from independent experiments were imaged for each condition. Mitochondrial ultrastructural features consistent with ferroptosis-like injury (e.g., mitochondrial shrinkage, increased membrane density, and reduced/disrupted cristae) were qualitatively evaluated based on representative images from multiple randomly selected fields.

### 2.12. Drug–Gene Interaction Analysis and Prioritization

We utilized three publicly available platforms—DGIdb (https://www.dgidb.org/, accessed on 18 January 2026), DrugBank (https://go.drugbank.com, accessed on 18 January 2026), and the Comparative Toxicogenomics Database (CTD, http://ctdbase.org/, accessed on 18 January 2026)—to identify potential drugs that directly or indirectly interact with the target genes. Drugs were prioritized based on interaction confidence, approval status, and reported relevance to neuroinflammation or iron metabolism.

### 2.13. Drug Intervention in Rotenone-Treated SH-SY5Y Cells (For qPCR Validation)

SH-SY5Y cells were pretreated for 1 h with clinically available compounds selected from the drug–gene interaction query, including N-acetylcysteine (NAC, 2 mM; Selleck Chemicals, Houston, TX, USA; Cat. No. S1623), orlistat (20 μM; Beyotime Biotechnology, Shanghai, China; Cat. No. SH0850), aspirin (1 mM; Beyotime Biotechnology, Shanghai, China; Cat. No. SH0768), or valproic acid (VPA, 1 mM; Selleck Chemicals, Houston, TX, USA; Cat. No. S3944), followed by co-exposure to rotenone (400 nM) for 24 h. NAC and VPA were prepared in sterile water; orlistat (and aspirin when needed) was prepared in DMSO. The final DMSO concentration was kept constant across all conditions (≤0.1%, *v*/*v*). After treatment, total RNA was extracted and subjected to qPCR analysis to assess *IL17A* (NAC), *LIPF* (orlistat), *GFAP* (aspirin), and *GATA3* (VPA). These compound concentrations were selected for short-term in vitro transcriptional-response assays and were not intended to model clinically achievable exposure levels.

## 3. Results

### 3.1. Network Toxicology Analysis Implicates Ferroptosis-Related Pathways in Rotenone Neurotoxicity

To explore the potential molecular mechanisms underlying rotenone-induced neurotoxicity, we first conducted a network toxicology analysis to predict putative targets of rotenone. Using SwissTargetPrediction and the Comparative Toxicogenomics Database (CTD), a total of 43 candidate targets were identified, which were primarily associated with redox regulation, mitochondrial function, and inflammatory signaling.

**Figure 1:**
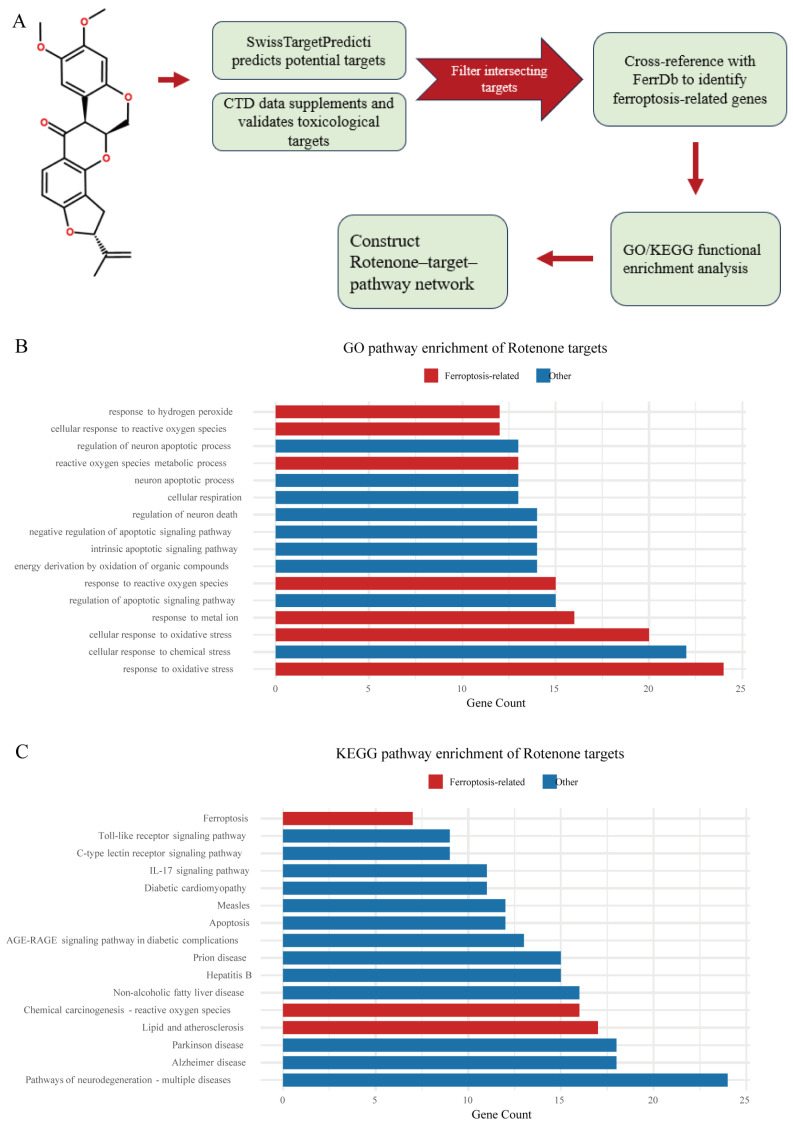
Network toxicology analysis predicts ferroptosis-related mechanisms of Rotenone. (**A**) Schematic workflow of network toxicology analysis. Predicted targets of Rotenone were obtained using SwissTargetPrediction and CTD, followed by intersection with ferroptosis-related genes from FerrDb. Functional enrichment (GO/KEGG) and network construction were then performed. GO enrichment analysis reveals Rotenone targets are mainly involved in redox homeostasis, lipid metabolism, and mitochondrial function. (**B**) KEGG pathway analysis highlights significant enrichment in ferroptosis and related oxidative stress pathways. (**C**) A compound–target–pathway interaction network was visualized using Cytoscape, emphasizing ferroptosis-relevant interactions in Rotenone’s toxicity profile.

By intersecting these predicted targets with curated ferroptosis-related gene sets from FerrDb and other databases, several overlapping genes with established roles in ferroptosis regulation were identified, including NRF2, TP53, GPX4, and ALOX5 (Figure 1A). Functional enrichment analysis of the predicted target set revealed significant associations with biological processes related to iron homeostasis, lipid metabolism, and oxidative stress responses (Figure 1B,C).

Collectively, these results indicate that ferroptosis-related pathways are engaged under rotenone exposure and provide a disease-contextual framework for subsequent transcriptomic and experimental validation.

### 3.2. Ferroptosis-Related Transcriptional Signatures in the Substantia Nigra of PD Patients

**Figure 2:**
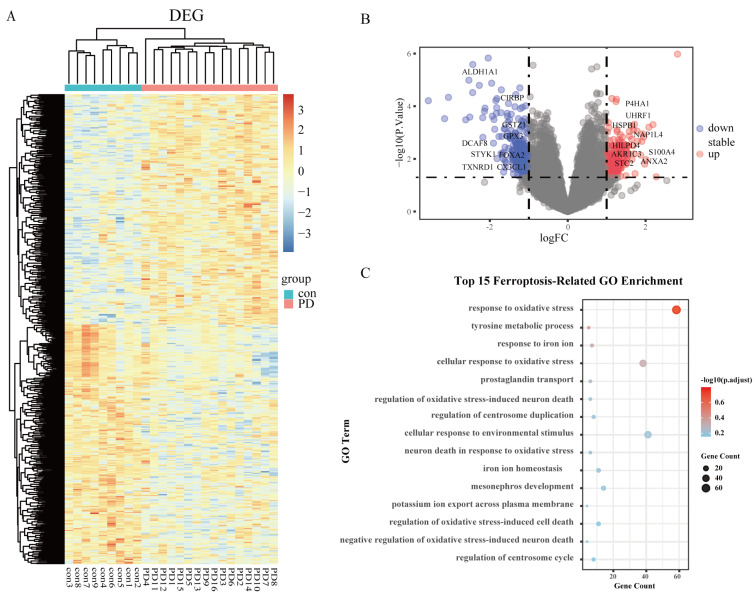
Ferroptosis-related transcriptional alterations in the substantia nigra of PD patients. (**A**) Heatmap showing differentially expressed genes between PD patients and healthy controls in the GSE7621 dataset. (**B**) Volcano plot of DEGs between PD patients and healthy controls in GSE7621. Red indicates upregulated genes and blue indicates downregulated genes; gray indicates non-significant genes. The dashed lines indicate the DEG thresholds of |log2FC| > 1 and adjusted *p* (FDR) < 0.05. Selected ferroptosis-related DEGs are annotated. (**C**) Bubble plot of the top 15 ferroptosis-related Gene Ontology biological processes enriched among the differentially expressed genes. Bubble size represents gene counts, and color indicates adjusted *p* (FDR).

To examine whether ferroptosis-related transcriptional alterations observed in PD patients are consistent with pathways implicated in rotenone neurotoxicity, we analyzed the GSE7621 dataset, which comprises substantia nigra samples from PD patients and matched healthy controls. A total of 281 genes were upregulated and 270 genes were downregulated in PD samples compared with controls (Figure 2A). Intersection with curated ferroptosis-related gene sets identified 21 differentially expressed ferroptosis-associated genes, including 12 upregulated genes and 9 downregulated genes (Figure 2B).

To determine whether ferroptosis-related biological processes are broadly altered in PD, Gene Ontology (GO) enrichment analysis was performed on all differentially expressed genes. Notably, a substantial subset of enriched GO terms was associated with ferroptosis-related processes, including iron homeostasis, lipid peroxidation, oxidative stress, and redox regulation. By systematically screening GO enrichment results using ferroptosis-relevant keywords, a total of 119 ferroptosis-associated biological processes were identified, indicating widespread perturbation of iron-dependent lipid oxidative pathways in the substantia nigra of PD patients (Supplementary Table S1). The top 15 most significantly enriched ferroptosis-related GO terms are shown in Figure 2C.

Together, these findings demonstrate that ferroptosis-related transcriptional programs are extensively dysregulated in PD, providing disease-contextual transcriptomic support for the involvement of ferroptosis-related mechanisms relevant to rotenone-associated neurodegenerative processes.

### 3.3. Ferroptosis-Related Transcriptional Heterogeneity and Network Organization in Parkinson’s Disease

To explore ferroptosis-related transcriptional heterogeneity in Parkinson’s disease, we analyzed substantia nigra transcriptomes from GSE7621. Using the expression profiles of 21 ferroptosis-related DEGs derived from PD-versus-control comparisons, PD samples were separated into two clusters by unsupervised consensus clustering (Supplementary Figure S2; see Section 2.3). To avoid over-interpretation, clusters were labeled neutrally as ferroptosis-signature-enriched versus ferroptosis-signature-depleted, reflecting relative expression differences rather than direct measurement of ferroptosis activity (Supplementary Figure S2E).

**Figure 3:**
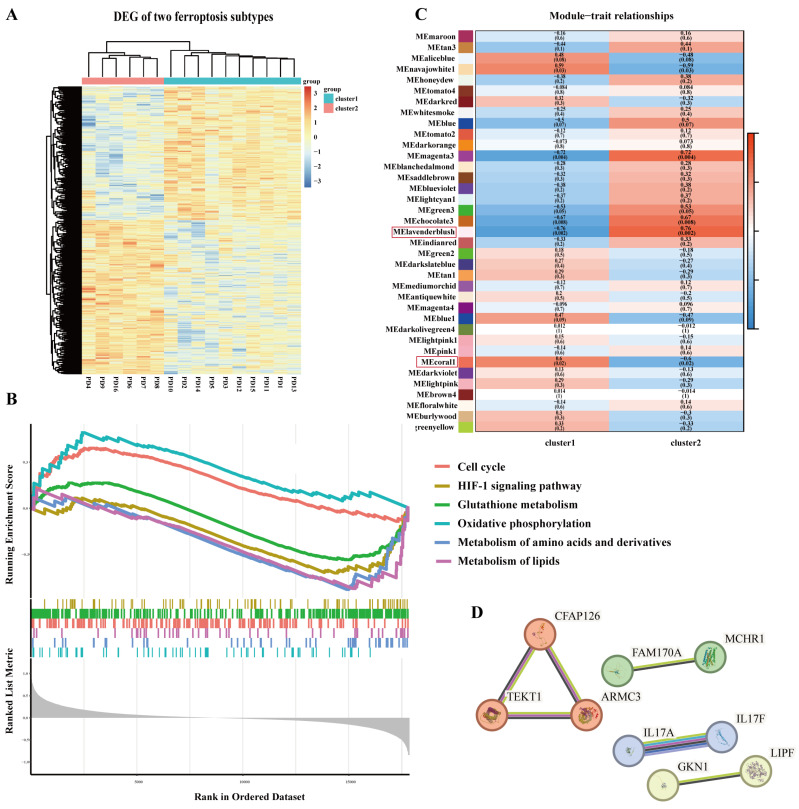
Ferroptosis-related transcriptional heterogeneity and network organization in Parkinson’s disease. (**A**) Heatmap showing subtype-associated transcriptional differences between Cluster 1 and Cluster 2 in PD substantia nigra samples from GSE7621, as defined by unsupervised clustering based on ferroptosis-related DEGs (Supplementary Figure S2). Clusters are described as ferroptosis-signature-enriched versus ferroptosis-signature-depleted states to reflect relative signature expression rather than a direct measurement of ferroptosis activity. (**B**) GSEA comparing Cluster 1 and Cluster 2, highlighting enrichment of ferroptosis- and mitochondrial/oxidative stress-related pathways in the ferroptosis-signature-enriched state. Different colors represent distinct enriched pathways. (**C**) WGCNA module–trait correlation analysis identifying co-expression modules most strongly positively and negatively associated with the ferroptosis-related signature/cluster trait. Red and blue colors indicate positive and negative module–trait correlations, respectively. Red boxes indicate the key WGCNA modules selected for downstream candidate gene prioritization. (**D**) PPI network constructed from the refined candidate set, highlighting the prioritized 11-gene hub set. This stratification is provided as a disease-context reference and should not be interpreted as evidence of rotenone exposure or toxin-specific causality. Note: This stratification is provided as a disease-context reference and is not interpreted as evidence of rotenone exposure or toxin-specific causality.

Differential expression analysis between these clusters identified 419 DEGs (Figure 3A), which were subsequently used to guide network-based analyses. This approach provides a disease-contextual reference for interpreting rotenone-exposed neuronal responses, without implying toxin-specific causality. The workflow is summarized in the schematic in Supplementary Figure S2A, highlighting that PD transcriptomic signatures are used solely as a contextual filter to prioritize candidate genes for downstream analysis. GSEA suggested preferential enrichment of ferroptosis- and mitochondrial/oxidative stress-related pathways in the ferroptosis-signature-enriched state (Figure 3B). WGCNA further identified modules most strongly positively and negatively associated with the ferroptosis-related signature/cluster trait (Figure 3C). Integrating these modules with subtype-associated DEGs yielded a refined candidate set, from which a PPI network was constructed and an 11-gene hub set was prioritized (Figure 3D). These analyses summarize ferroptosis-associated transcriptional organization in PD, providing a disease-contextual reference for interpreting rotenone-exposed neuronal responses, without implying direct toxin-specific causality.

### 3.4. Functional Validation Confirms Ferroptosis Involvement in Rotenone-Induced Neurotoxicity

**Figure 4:**
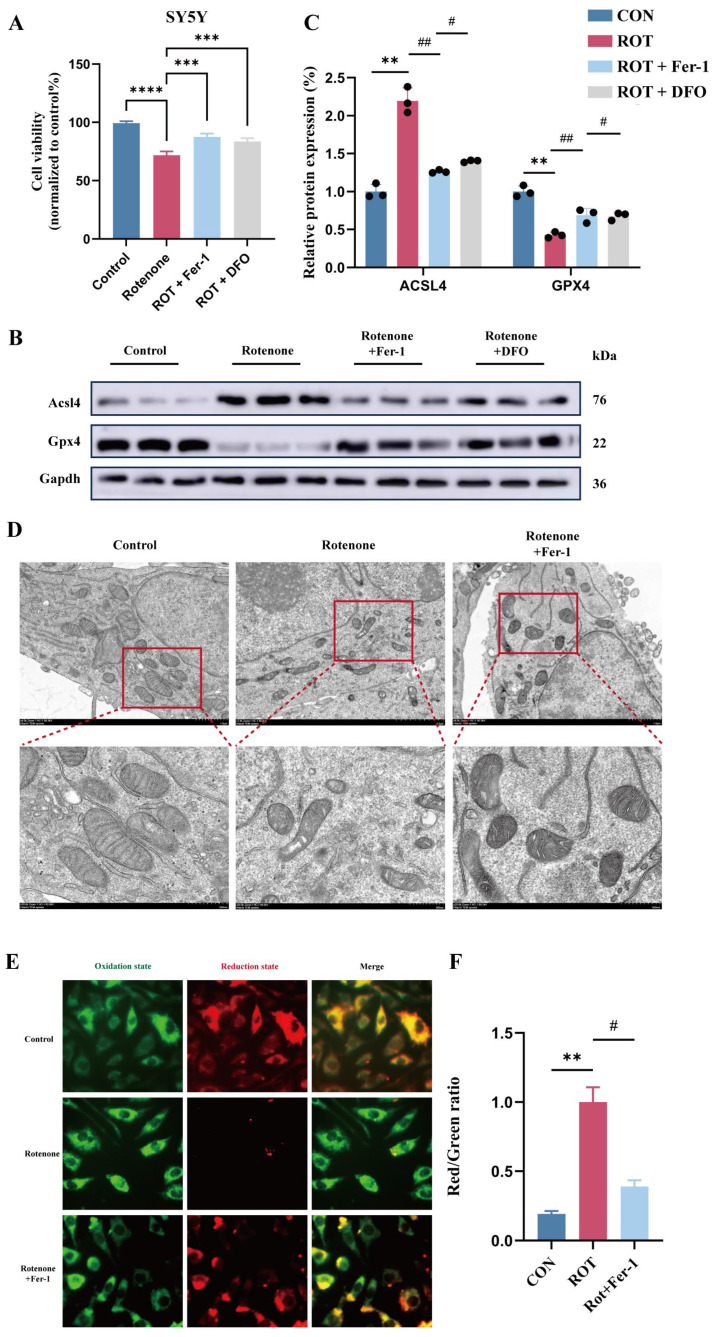
Functional validation of ferroptosis involvement in rotenone-induced neurotoxicity. (**A**) CCK-8 assay showing reduced cell viability after rotenone treatment and its partial rescue by Fer-1 and DFO. (**B**) Representative Western blot analysis of GPX4 and ACSL4 expression under the indicated treatments. (**C**) Densitometric quantification of GPX4 and ACSL4 protein levels normalized to GAPDH. (**D**) Representative transmission electron microscopy images showing ferroptosis-like mitochondrial alterations induced by rotenone and their attenuation by Fer-1. (**E**) Lipid peroxidation detected by C11-BODIPY 581/591 fluorescence staining. Green fluorescence indicates the oxidized form of the probe, red fluorescence indicates the reduced form, and merged images show the overlap of oxidized and reduced signals. (**F**) Quantification of lipid peroxidation based on C11-BODIPY fluorescence intensity. Data are presented as mean ± SD from three independent experiments. Statistical significance was determined by one-way ANOVA with post hoc multiple-comparison testing. ** *p* < 0.01, *** *p* < 0.001, and **** *p* < 0.0001 vs. control; # *p* < 0.05 and ## *p* < 0.01 vs. rotenone.

To functionally validate the involvement of ferroptosis in rotenone-induced neurotoxicity, a series of phenotypic, molecular, and ultrastructural assays were performed in SH-SY5Y cells. CCK-8 assays showed that rotenone treatment significantly reduced cell viability compared with control cells, whereas co-treatment with the ferroptosis inhibitor Fer-1 markedly attenuated this cytotoxic effect. Similarly, the iron chelator DFO partially restored cell viability, indicating an iron-dependent cell death mechanism (Figure 4A).

At the molecular level, Western blot analysis and densitometric quantification showed that rotenone exposure decreased GPX4 and increased ACSL4, whereas Fer-1 and DFO substantially reversed these protein alterations (Figure 4B,C).

Transmission electron microscopy (TEM) provided representative ultrastructural evidence that rotenone induced mitochondrial damage (mitochondrial shrinkage, increased membrane electron density, and cristae disruption), which appeared less pronounced with Fer-1 co-treatment (Figure 4D). Lipid peroxidation analysis using C11-BODIPY 581/591 fluorescence showed increased oxidized fluorescence in rotenone-treated cells, which was quantified as Red/Green ratio and effectively suppressed by Fer-1 (Figure 4E,F). Collectively, these results provide convergent functional, molecular, and morphological evidence that ferroptosis contributes to rotenone-induced neuronal toxicity.

### 3.5. Ferroptosis Inhibition Attenuates Rotenone-Induced Upregulation of a Network-Derived Candidate Transcriptional Module

**Figure 5:**
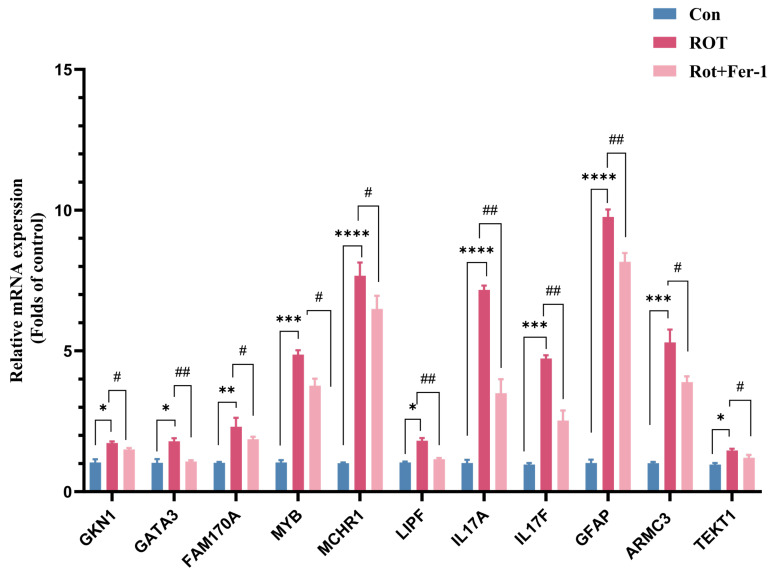
Ferrostatin-1 treatment attenuates rotenone-induced upregulation of a network-derived ferroptosis-associated candidate module. Quantitative real-time PCR (qPCR) analysis of an 11-gene, network-derived ferroptosis-associated candidate module (*LIPF*, *FAM170A*, *MCHR1*, *IL17A*, *MYB*, *GFAP*, *ARMC3*, *GKN1*, *GATA3*, *IL17F*, and *TEKT1*) in SH-SY5Y cells treated with rotenone (400 nM, 24 h) alone or co-treated with the ferroptosis inhibitor ferrostatin-1 (Fer-1, 2 µM). Rotenone significantly increased the mRNA levels of all 11 candidates relative to untreated controls (* *p* < 0.05, ** *p* < 0.01, *** *p* < 0.001, and **** *p* < 0.0001 vs. control). Co-treatment with Fer-1 significantly reduced their expression compared with rotenone alone (# *p* < 0.05, ## *p* < 0.01 vs. rotenone). Data are presented as mean ± SD (*n* = 3 independent experiments).

To further examine whether rotenone-induced upregulation of this network-derived candidate transcriptional module is linked to ferroptosis-associated stress, SH-SY5Y cells were co-treated with rotenone and the ferroptosis inhibitor ferrostatin-1 (Fer-1). qPCR analysis showed that rotenone significantly increased the expression of all 11 candidates, whereas Fer-1 co-treatment significantly attenuated their induction (Figure 5). In particular, inflammation- and stress-associated genes (*IL17A*, *IL17F*, *GFAP*, and *GATA3*) displayed the strongest Fer-1-sensitive reductions. Genes related to lipid metabolism and oxidative stress responses (e.g., *LIPF* and *GKN1*) also exhibited partial normalization. The remaining candidates implicated in cellular structure, transcriptional regulation, and neuronal homeostasis (*FAM170A*, *ARMC3*, *TEKT1*, *MYB*, and *MCHR1*) showed more heterogeneous but still significant attenuation with Fer-1. Together, these results indicate that activation of this 11-gene panel under rotenone exposure is at least partly Fer-1-sensitive, consistent with a ferroptosis-linked transcriptional response and supporting their interpretation as ferroptosis-responsive transcriptional markers, rather than direct mediators of ferroptotic cell death.

### 3.6. Exploratory Drug–Gene Interaction Analysis

To explore whether the network-derived hub module could be pharmacologically interrogated, we queried DGIdb, DrugBank, and CTDbase for reported drug–gene associations involving the hub genes. This analysis was used for hypothesis generation and prioritization, with full interaction records provided in Supplementary Table S2.

As a limited expression-level check, selected compounds were tested in rotenone-treated SH-SY5Y cells. qPCR analysis showed partial attenuation of representative hub-gene induction (Supplementary Figure S3). These data were interpreted as exploratory transcript-level readouts rather than evidence of target engagement, functional protection, or therapeutic efficacy.

## 4. Discussion

Rotenone is a naturally occurring botanical pesticide widely used in agriculture and aquaculture [28]. Owing to its environmental persistence and lipophilicity, rotenone can penetrate biological membranes and accumulate in non-target organisms, raising long-standing concerns regarding its neurotoxic risk in humans and wildlife [29,30,31]. Mechanistically, rotenone inhibits mitochondrial complex I and promotes oxidative stress, thereby producing dopaminergic injury and PD-like motor phenotypes [29]. Increasing evidence supports the involvement of ferroptosis—an iron-dependent, lipid peroxidation-driven form of regulated cell death—in rotenone-induced neuronal injury. Our study strengthens and contextualizes these observations within a human PD transcriptomic framework. [32,33]. However, prior studies have often relied on limited or correlative readouts; from a toxicology perspective, more specific functional evidence (e.g., ferroptosis inhibition/iron chelation rescue together with lipid peroxidation and molecular/ultrastructural endpoints) remains important to define the contribution of ferroptosis to rotenone neurotoxicity and to interpret it within a disease-contextual molecular network framework [34,35].

In this study, we applied a network toxicology approach integrating SwissTargetPrediction and the CTD to predict 43 putative rotenone-associated targets, followed by enrichment analysis that highlighted biological processes intersecting ferroptosis-relevant pathways, including iron handling/transport, lipid metabolism, and oxidative stress regulation. To further place these predicted processes in a clinically relevant context, we cross-referenced them with transcriptomic data from the substantia nigra of PD patients (GSE7621). Notably, although rotenone is frequently used to establish experimental PD models, the human PD transcriptomic dataset used here does not derive from rotenone exposure; rather, it serves as a clinical reference to evaluate whether the rotenone-predicted ferroptosis-relevant programs are also manifested in diseased human tissue. The ferroptosis-associated signals observed in PD provide translational plausibility for this toxicological hypothesis; importantly, we subsequently tested this hypothesis in a direct rotenone exposure model using functional ferroptosis assays.

By analyzing transcriptomic data from human PD substantia nigra tissues, we first stratified PD samples into two transcriptomic clusters (Cluster 1 and Cluster 2) using unsupervised clustering based on the expression of ferroptosis-related DEGs. To avoid over-interpretation, we describe these clusters as ferroptosis-signature-enriched versus ferroptosis-signature-depleted states, reflecting relative differences in signature expression rather than a direct measurement of ferroptosis activity. Such exploratory molecular stratification approaches have been shown to be valuable for uncovering disease heterogeneity [36]. Building on this stratification, differential expression analysis combined with weighted gene co-expression network analysis (WGCNA) converged on an 11-gene hub set (*LIPF*, *FAM170A*, *MCHR1*, *IL17A*, *MYB*, *GFAP*, *ARMC3*, *GKN1*, *GATA3*, *IL17F*, and *TEKT1*). Collectively, these genes were prioritized through PD network convergence and exhibited Fer-1-sensitive normalization under rotenone exposure, supporting their interpretation as a ferroptosis-responsive transcriptional signature in PD-relevant toxicant paradigms. However, they should not be interpreted as direct mediators of ferroptotic cell death, because causal roles have not yet been tested by gene-specific perturbation.

To establish a functional context for interpreting this network-derived module, we next performed ferroptosis-focused assays in rotenone-treated SH-SY5Y cells, which yielded results consistent with a ferroptosis-associated injury state that was pharmacologically suppressible by Fer-1 and/or DFO. Against this backdrop, we asked whether the 11 hub genes behave as a Fer-1-sensitive transcriptional signature by examining their sensitivity to Fer-1 co-treatment.

In the context of PD pathology, this 11-gene hub set can be linked to processes involving inflammation–oxidative stress coupling, glial reactivity, iron homeostasis, and lipid peroxidation. IL17A/IL17F are associated with inflammatory programs that have been connected to iron-related stress responses and may shape susceptibility to iron-dependent injury [37,38]. Upregulation of GFAP reflects reactive astrocyte activation and indicates enhanced glial responses in the PD substantia nigra, commonly associated with oxidative stress [11]. From a toxicological perspective, rotenone exposure is characterized by iron dyshomeostasis, disruption of glutathione metabolism, and accumulation of lipid ROS [36,38]. These events provide a coherent framework for interpreting the hub genes: iron-related stress can be discussed alongside IL-17 signaling and GFAP-associated glial responses [39,40,41]; glutathione metabolic disturbance may involve transcriptional regulators such as MYB and GATA3 in oxidative stress control [42,43]; and lipid oxidative pressure can be considered in relation to lipid metabolism- or signaling-related nodes, including LIPF (a lipid-peroxidation-linked gene), MCHR1, and ARMC3 [44]. In addition, the precise roles of FAM170A, TEKT1, and GKN1 remain unclear, but these genes may participate in cross-regulatory processes involving cytoskeletal organization, signal transduction, and oxidative stress, potentially adding an additional layer of regulation relevant to ferroptosis-associated injury [45,46].

At the functional level, Fer-1 co-treatment was used as a reference to assess whether this hub module exhibits a ferroptosis-associated transcriptional response. IL17A, IL17F, GATA3, and GFAP showed the strongest responsiveness to Fer-1. IL17A/IL17F/GATA3 are closely linked to inflammatory and stress-related signaling, consistent with emerging evidence that lipid oxidation products generated during ferroptosis can amplify pro-inflammatory signaling in neural injury contexts [37,38]. Concurrently, GFAP upregulation is commonly interpreted as a marker of astrocyte activation, and astrocyte-associated oxidative stress and disrupted iron homeostasis have been implicated in the aggravation of neuronal injury [10,47,48]. Moreover, Fer-1-sensitive modulation of lipid- and stress-associated genes such as LIPF and GKN1 aligns with the central role of lipid remodeling and redox adaptation in determining ferroptosis susceptibility [11,46]. Although the remaining candidates (FAM170A, ARMC3, TEKT1, MYB, and MCHR1) displayed more heterogeneous expression patterns, their consistent Fer-1 responsiveness suggests involvement in adaptive transcriptional regulation downstream of ferroptosis-related stress rather than direct execution of ferroptotic cell death [44,45,49,50]. Collectively, these genes were prioritized through PD network convergence and exhibited Fer-1-dependent normalization under rotenone exposure, supporting their interpretation as a ferroptosis-associated transcriptional signature in PD-relevant toxicant paradigms; however, distinguishing causal regulators from responsive markers will require further perturbation-based mechanistic studies.

Our drug–gene interaction component is intended as a forward-looking prioritization step rather than evidence of therapeutic efficacy. Because DGIdb, DrugBank, and CTDbase aggregate heterogeneous evidence types, including curated interactions, pathway associations, and literature co-mentions, database-derived hits should not be equated with confirmed target engagement in the rotenone model. To avoid over-interpretation and reduce distraction from the primary ferroptosis–rotenone narrative, the detailed interaction records are provided in Supplementary Table S2, and the limited qPCR validation has been moved to Supplementary Figure S2. These data were interpreted only as exploratory transcript-level readouts showing that selected compounds can modulate representative hub-gene expression under rotenone-induced stress. Because these compounds were not specific ferroptosis inhibitors, they were evaluated only at the transcript level, and included relatively high in vitro concentrations of aspirin and valproic acid. The findings should not be interpreted as evidence of clinically relevant neuroprotection, target engagement, ferroptosis suppression, or therapeutic efficacy. Further dose–response and viability-based studies, ideally benchmarked against ferroptosis-focused controls such as Fer-1 and DFO, will be required before any functional or therapeutic conclusions can be drawn.

In summary, by integrating network toxicology with human PD substantia nigra transcriptomic context and targeted functional validation, this study supports ferroptosis as a mechanistically relevant component of rotenone-induced PD-like neurotoxicity. Beyond establishing a ferroptosis-associated injury state in rotenone-exposed neurons, our analyses nominate an 11-gene, PD-contextualized hub module that is recapitulated under rotenone exposure and shows Fer-1-sensitive normalization, suggesting its potential utility as a ferroptosis-responsive transcriptional signature for hypothesis-driven mechanistic testing, rather than evidence that these genes directly mediate ferroptotic cell death. These findings primarily provide a disease-informed toxicological framework and a focused set of hypotheses for mechanistic interrogation rather than evidence for clinical intervention.

Several limitations should be acknowledged. First, the PD transcriptomic dataset was not derived from rotenone exposure and was used as a disease-context reference; therefore, overlap with rotenone responses does not establish causality. Second, ferroptosis-related gene sets and keyword-based pathway screening are constrained by current annotations and may introduce selection bias. Third, our in vitro validation was performed in a single SH-SY5Y cell model and was based primarily on pharmacological and phenotypic criteria, including Fer-1/DFO rescue, GPX4/ACSL4 modulation, lipid peroxidation, and mitochondrial ultrastructural changes. Although these assays provide convergent support for ferroptosis-associated injury, genetic perturbation experiments, such as GPX4 knockdown or overexpression, were not included. Future studies incorporating genetic ferroptosis modulation and additional neuronal models will be important to further define causality and cell-type generalizability.

## 5. Conclusions

By integrating network toxicology and PD substantia nigra transcriptomics, this study prioritized ferroptosis-relevant programs and a PD-contextualized, ferroptosis-associated candidate module linked to rotenone neurotoxicity. Functional assays in rotenone-exposed SH-SY5Y cells supported a ferroptosis-associated injury state that could be attenuated by ferroptosis inhibition or iron chelation. The 11-gene module should therefore be interpreted as a ferroptosis-responsive transcriptional signature for hypothesis-driven mechanistic testing, rather than as direct evidence that these genes mediate ferroptotic cell death.

## Figures and Tables

**Table 1:** Sequences of primers used in real-time PCR analysis.

Gene	Forward Primer	Reverse Primer
*LIPF*	5′-TTGGACCCAGGCTGTTAAGTC-3′	5′-TTGTAGTAGGGAGGTTGGGAC-3′
*GKN1*	5′-CTGTCCACTGCTTTCGTGAAG-3′	5′-GTCCCATCCGTTGTTATTGTCAA-3′
*MCHR1*	5′-ATGGATCTGCAAGCCTCGTTG-3′	5′-CACGACCGCGAAGATGACC-3′
*FAM170A*	5′-TGTCTCCTTGTCGTCCTATTCA-3′	5′-GGAGTACCTACCCTCACAACC-3′
*MYB*	5′ATCTCCCGAATCGAACAGATGT-3′	5′-TGCTTGGCAATAACAGACCAAC-3′
*IL17F*	5′-GCTGTCGATATTGGGGCTTG-3′	5′-GGAAACGCGCTGGTTTTCAT-3′
*IL17A*	5′-TCCCACGAAATCCAGGATGC-3′	5′-GGATGTTCAGGTTGACCATCAC-3′
*GATA3*	5′-GCCCCTCATTAAGCCCAAG-3′	5′-TTGTGGTGGTCTGACAGTTCG-3′
*GFAP*	5′-AGGCTACACTCAAATTATTGCCA-3′	5′-CTTGCCTAAGATTTCAGGACACA-3′
*TEKT1*	5′-GGCACATTGCTAACAAGAACCA-3′	5′-CTCTGGCTTTCTGCGACCA-3′
*ARMC3*	5′-GGATTAGAGCCACTCATCAGAC-3′	5′-GCCAACAACTGAATCACTGGAT-3′

## Data Availability

The original contributions presented in this study are included in the article/Supplementary Material. Further inquiries can be directed to the corresponding authors.

## Supplementary Materials

The following supporting information can be downloaded at: https://www.mdpi.com/article/10.3390/cells15110959/s1, Supplementary Table S1: Full list of ferroptosis-associated GO biological processes enriched in the GSE7621 PD differential expression analysis; Supplementary Table S2: Predicted drug–gene interactions for ferroptosis-related genes, with supporting references included in the main reference list [39,49,51,52,53,54,55,56,57,58,59,60,61,62,63,64,65]; Supplementary Figure S1: Unsupervised stratification of PD substantia nigra samples based on ferroptosis-related DEGs in GSE7621; Supplementary Figure S2: Limited expression-level validation of selected clinically available compounds on representative hub-gene transcripts under rotenone exposure; Supplementary Figure S3: Dose–response analysis of rotenone-induced cytotoxicity in SH-SY5Y cells measured by CCK-8 assay.

## Abbreviations

**Abbreviation**	**Full Term**
PD	Parkinson’s disease
DEGs	Differentially expressed genes
ROS	Reactive oxygen species
FDR	False discovery rate
FC	Fold change
GEO	Gene Expression Omnibus
GSE	Gene Expression Omnibus Series
WGCNA	Weighted gene co-expression network analysis
PPI	Protein–protein interaction
GO	Gene Ontology
KEGG	Kyoto Encyclopedia of Genes and Genomes
GSEA	Gene set enrichment analysis
qPCR	Quantitative polymerase chain reaction
CTD	Comparative Toxicogenomics Database
DGIdb	Drug–Gene Interaction Database
NAC	N-acetylcysteine
VPA	Valproic acid
Fer-1	ferrostatin-1
CCK-8	Cell viability assay
DFO	Deferoxamine
TEM	Transmission electron microscopy
CDF	cumulative distribution function
