# Supplementary material for: Network Toxicology and Transcriptomic Analyses Reveal Ferroptosis-Related Neurotoxicity of Rotenone as an Environmental Hazardous Compound
Source: Cells. 2026 May 22;15(11):959. doi: 10.3390/cells15110959 (PMC13256928; doi:10.3390/cells15110959)
Supplement: Supplementary file 1 [file cells-15-00959-s001.zip › Supplementary Figure S1-S3.docx]

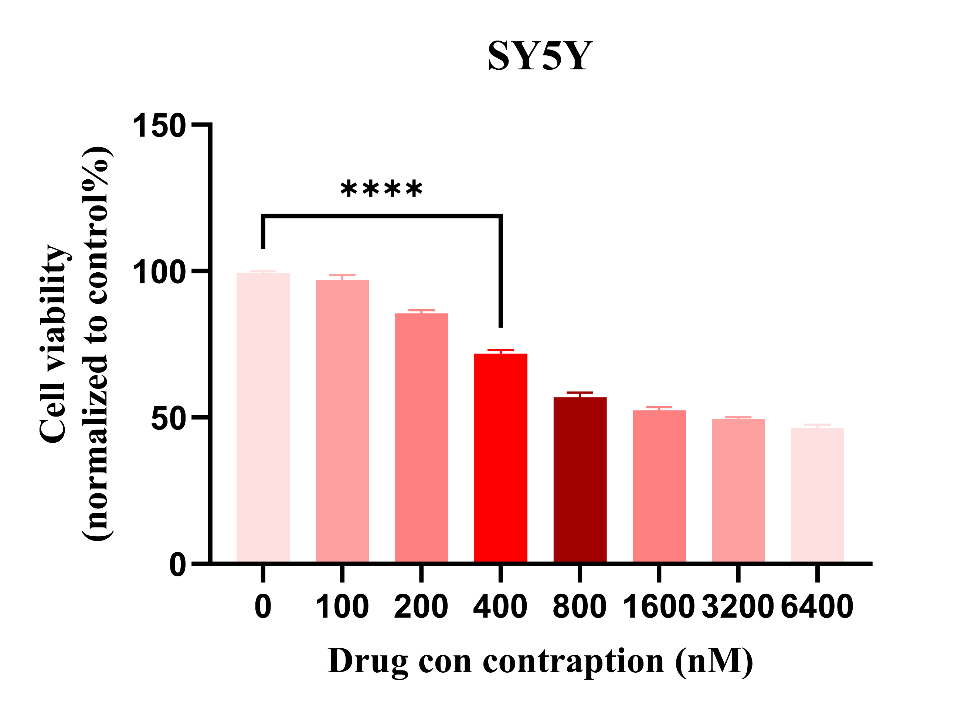


Supplementary Figure S1. Dose–response of rotenone-induced cytotoxicity measured by CCK-8 in SH-SY5Y cells. SH-SY5Y cells were treated with rotenone at the indicated concentrations (0, 100, 400, and 800 nM) for 24 h, followed by CCK-8 assay to quantify cell viability. Viability is expressed as a percentage of the vehicle control group (0 nM). The vehicle concentration was kept constant across all groups (final DMSO ≤ 0.1%, v/v). Data are presented as mean ± SD from three independent experiments. Statistical significance was determined by one-way ANOVA with post hoc multiple-comparison testing. *P < 0.05 and **P < 0.01 vs. control.

Supplementary Results 2. Rationale and procedure for ferroptosis-related subtyping of PD samples in GSE7621

To understand how PD samples were stratified, we performed unsupervised consensus clustering on substantia nigra transcriptomes from GSE7621, using the 21 ferroptosis-related DEGs identified from the PD-versus-control comparison (see Methods). Consensus clustering was run with the PAM algorithm and Euclidean distance across k = 2–9, with 80% of samples resampled 1000 times to ensure stability.

A schematic overview of this workflow is provided in Supplementary Fig. S2A, illustrating how PD transcriptomic data were used as a disease-context reference for prioritizing candidate modules. The volcano plot highlighting the 21 ferroptosis-related DEGs is shown in Supplementary Fig. S1B. Across k values, k = 2 provided a clear and stable separation, as reflected in the consensus matrix heatmap (Supplementary Fig. S2C). tSNE visualization confirmed the two-cluster assignment (Supplementary Fig. S2D), and a heatmap of the 21-gene signature showed consistent expression differences between Cluster 1 and Cluster 2 (Supplementary Fig. S2E).

To avoid over-interpretation, we labeled the clusters neutrally as ferroptosis-signature–enriched versus ferroptosis-signature–depleted, reflecting relative expression differences rather than direct measures of ferroptosis activity. Importantly, these PD samples are from clinical tissue, not rotenone-exposed cells; therefore, this stratification should be interpreted as a disease-contextual reference to guide downstream analyses and hypothesis generation, not as evidence of rotenone-specific or toxin-specific effects.


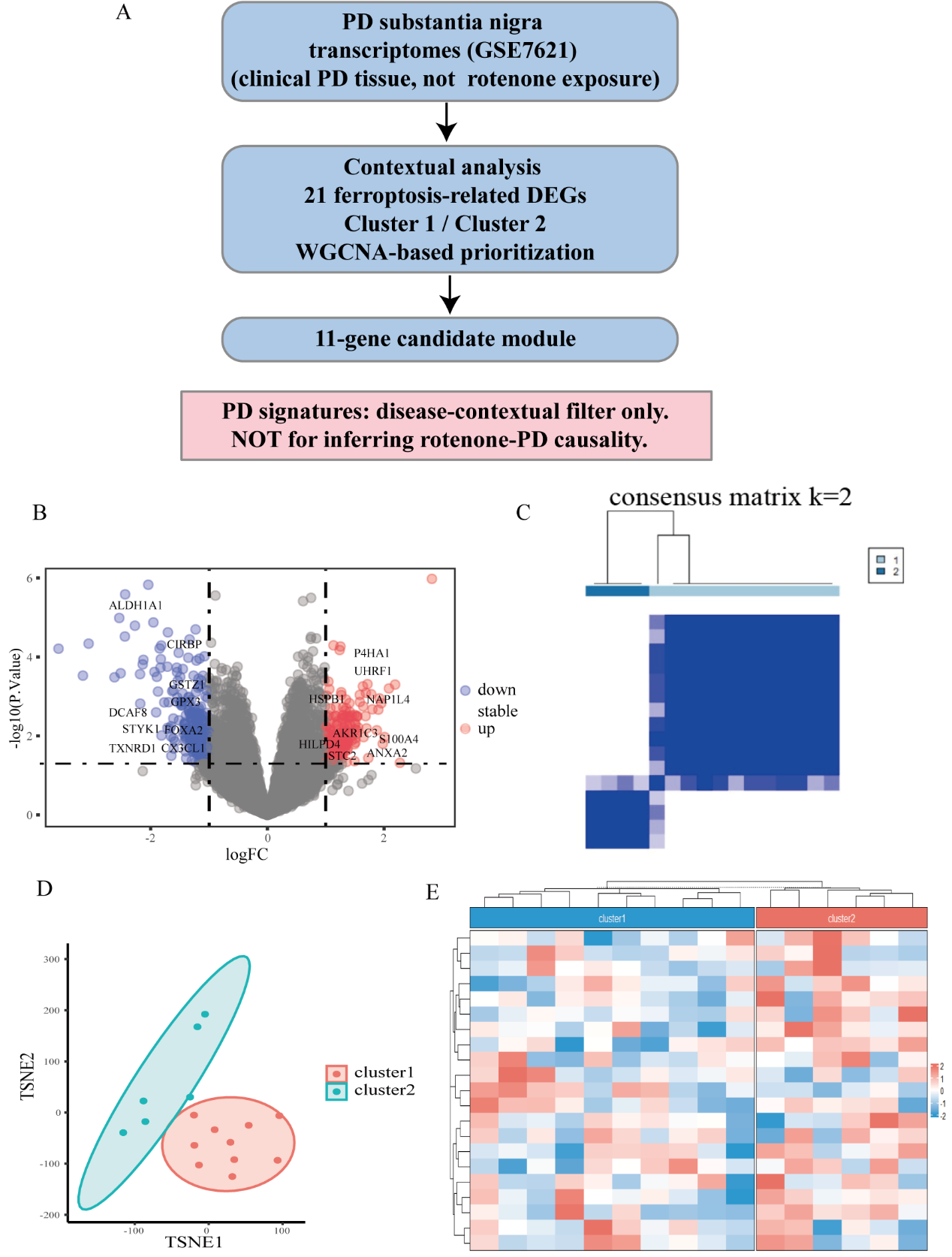


Supplementary Figure S2. Unsupervised stratification of PD substantia nigra samples based on ferroptosis-related DEGs (GSE7621).

(A) Schematic showing how PD transcriptomic data were used as a disease-context reference to guide candidate prioritization.

(B) Volcano plot of DEGs between PD patients and healthy controls, highlighting the 21 ferroptosis-related DEGs used as input for clustering.

(C) Consensus matrix heatmap (k = 2), showing high within-cluster agreement and low between-cluster mixing.

(D) tSNE visualization of PD samples colored according to the two clusters.

(E) Heatmap of the 21 ferroptosis-related DEGs across PD samples, showing distinct expression patterns between Cluster 1 and Cluster 2. Clusters are labeled as ferroptosis-signature–enriched versus ferroptosis-signature–depleted to indicate relative signature expression rather than direct ferroptosis measurement.

Note: These clusters provide a disease-context reference for downstream analyses and do not indicate rotenone-specific or toxin-specific effects.


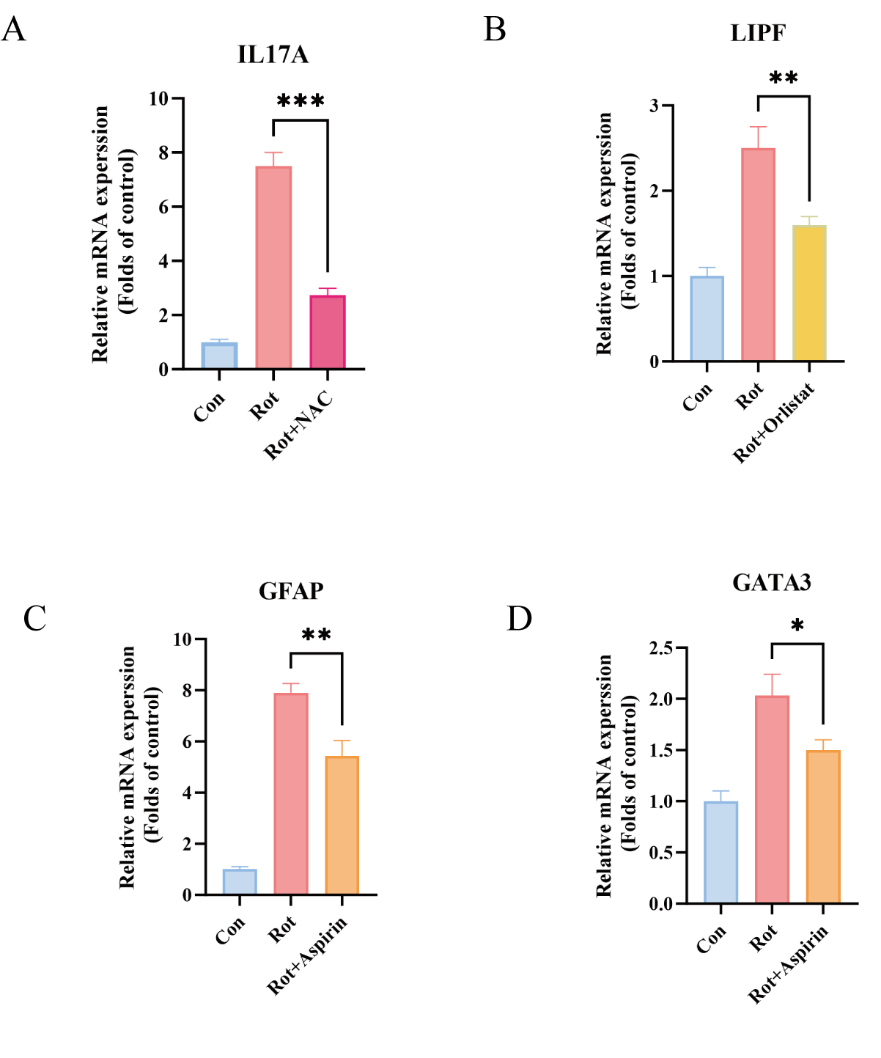


Supplementary Figure S3. Limited expression-level validation of selected clinically available compounds on representative hub-gene transcripts under rotenone exposure. SH-SY5Y cells were exposed to rotenone and treated with the indicated compounds. qPCR was used to assess transcript changes of representative network-derived candidates: IL17A (rotenone ± N-acetylcysteine, NAC), LIPF (rotenone ± orlistat), GFAP (rotenone ± aspirin), and GATA3 (rotenone ± valproic acid, VPA). The drug–gene associations were initially prioritized by database queries (DGIdb/DrugBank/CTDbase; see Supplementary Table S2) and are presented here as exploratory transcript-level readouts rather than evidence of target engagement, functional neuroprotection, or therapeutic efficacy. Data are shown as mean ± SD (n = 3). *P < 0.05, **P < 0.01 vs. rotenone.
