# Supplementary material for: Network Toxicology and Transcriptomic Analyses Reveal Ferroptosis-Related Neurotoxicity of Rotenone as an Environmental Hazardous Compound
Source: Cells. 2026 May 22;15(11):959. doi: 10.3390/cells15110959 (PMC13256928; doi:10.3390/cells15110959)
Supplement: Supplementary file 1 [file cells-15-00959-s001.zip › Supplementary Table S2.docx]

Supplementary Table S2. Predicted drug–gene interactions for ferroptosis-related genes

Note: Drug–gene interactions were retrieved from DGIdb/DrugBank/CTDbase and represent heterogeneous evidence types (e.g., curated interactions, pathway-level associations, and literature co-mentions). These entries were not interpreted as confirmed target engagement and are presented for hypothesis generation only. Where pharmacology is ambiguous or not directly supported (e.g., antipsychotics), entries were deprioritized and not used for experimental selection.

| **Gene** | **Drug** | **Reported pharmacology** | **Clinical Stage** | **Structure** |
| --- | --- | --- | --- | --- |
| GATA3 | Tamoxifen[1] | Indirectly upregulates GATA3 expression | Approved | 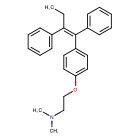 |
| GATA3 | Valproic Acid[2] | Increases GATA3 via chromatin modulation | Approved | 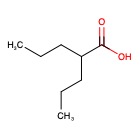 |
| GATA3 | Resveratrol[3] | Modulates GATA3-related pathways | Preclinical  /Supplement | 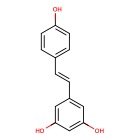 |
| GATA3 | SB010 DNAzyme[4] | Cleaves GATA3 mRNA to suppress its expression | Phase II trial | 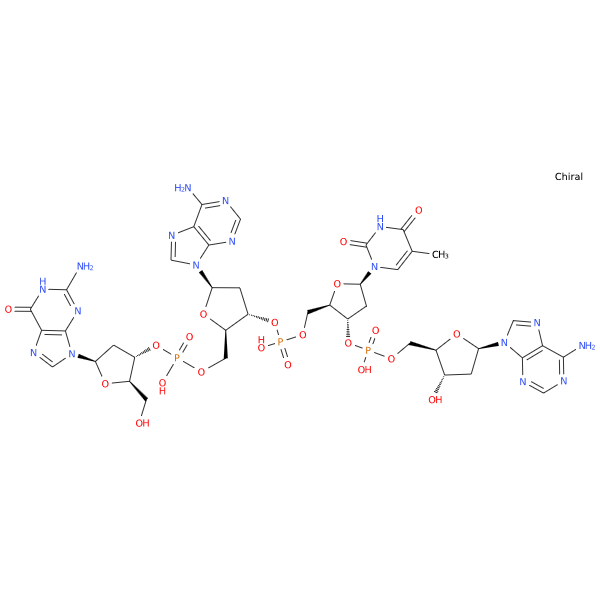 |
| GFAP | Aspirin[5] | Reduces astrocyte activation and GFAP levels | Approved | 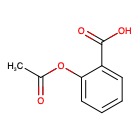 |
| LIPF | Orlistat[6] | Irreversible inhibition of gastric/pancreatic lipases, reducing free fatty acid generation | FDA approved | 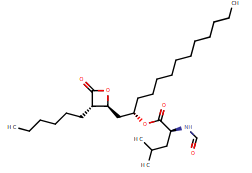 |
| MCHR1 | AMG 076[7] | Selective antagonists of MCHR1, suppressing appetite and energy intake | Preclinical | 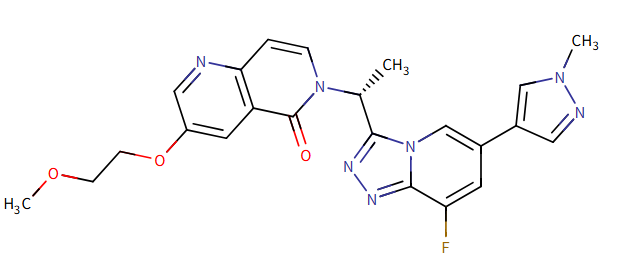 |
| MCHR1 | NGD-4715[8] | Selective antagonists of MCHR1 | Preclinical | 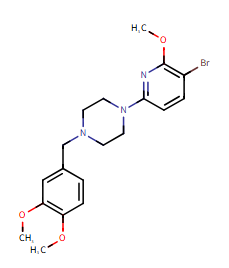 |
| GFAP | Tretinoin[9] | Induces astrocytic differentiation, affecting GFAP expression | FDA approved | 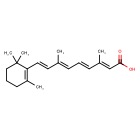 |
| GFAP | 15d-PGJ 2[10] | Inhibits inflammatory responses in astrocytes, downregulating GFAP | Preclinical | 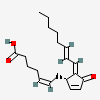 |
| GFAP | Minocycline[11] | Attenuates gliosis and GFAP upregulation | Phase IV  /Repurposed | 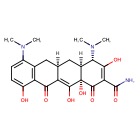 |
| MYB | 5-FU[12] | chemotherapeutic antimetabolite | FDA approved | 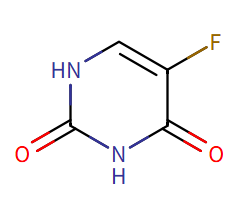 |
| MYB | Paclitaxel[13] | microtubule stabilizer | FDA approved | 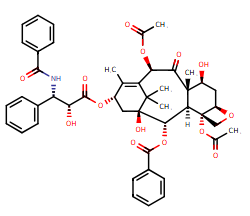 |
| MYB | Doxorubicin[14] | topoisomerase II inhibitor | FDA approved | 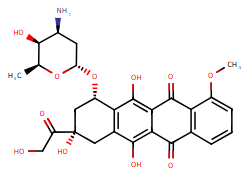 |
| IL17A | Secukinumab[15] | Neutralizes IL-17A cytokine, blocking its signaling | FDA approved | 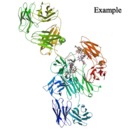 |
| IL17A | Ixekizumab[16] | Binds and neutralizes IL-17A, preventing inflammation | FDA approved | 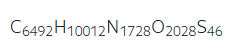 |
| IL17A | Bimekizumab[17] | Neutralizes IL-17A and IL-17F, broad IL-17 pathway inhibition | Phase III  /Approved | 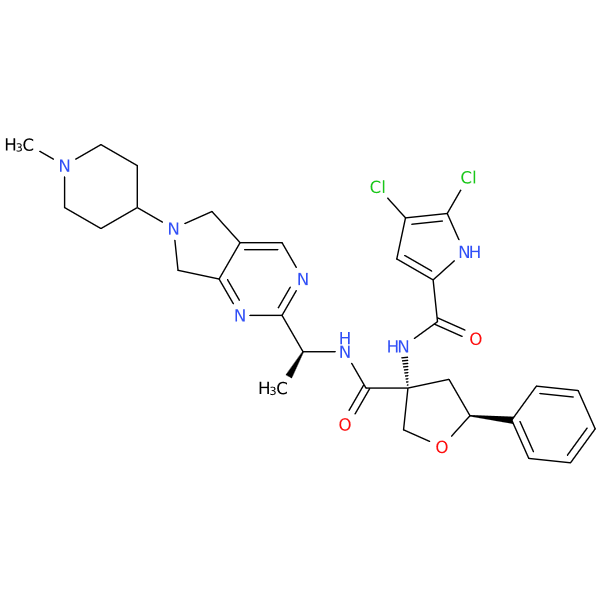 |
| IL17A | N-acetylcysteine[18] | Lowers IL-17A levels via reducing oxidative inflammation | FDA approved | 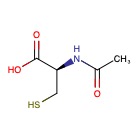 |
| IL17F | Bimekizumab[17] | Neutralizes both IL-17A and IL-17F | Phase III | 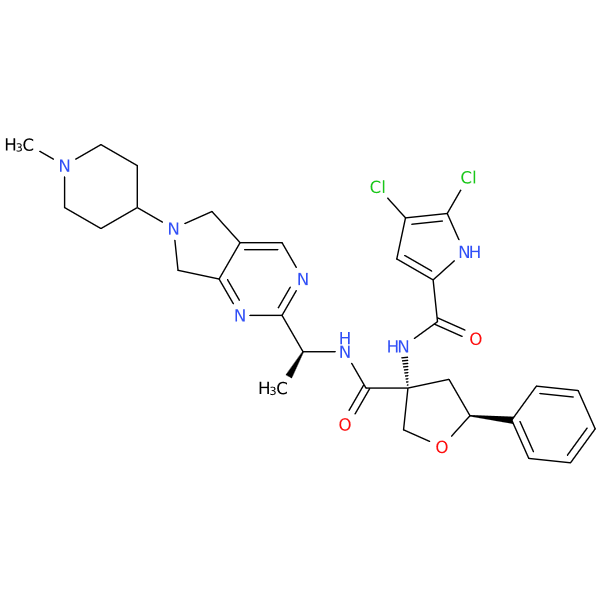 |
| IL17F | Ustekinumab[19] | IL-12/23 p40 antibody antagonist | FDA approved | 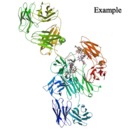 |

**References**

1. Sandström, J.; Bomanson, J.; Pérez-Tenorio, G.; Jönsson, C.; Nordenskjöld, B.; Fornander, T.; Lindström, L.S.; Stål, O. GATA3 and markers of epithelial-mesenchymal transition predict long-term benefit from tamoxifen in ER-positive breast cancer. *NPJ Breast Cancer* **2024**, *10*, 78, doi:10.1038/s41523-024-00688-6.

2. Rout, U.K.; Clausen, P. Common increase of GATA-3 level in PC-12 cells by three teratogens causing autism spectrum disorders. *Neurosci Res* **2009**, *64*, 162-169, doi:10.1016/j.neures.2009.02.009.

3. Bakheet, S.A.; Alzahrani, M.Z.; Ansari, M.A.; Nadeem, A.; Zoheir, K.M.A.; Attia, S.M.; Al-Ayadhi, L.Y.; Ahmad, S.F. Resveratrol Ameliorates Dysregulation of Th1, Th2, Th17, and T Regulatory Cell-Related Transcription Factor Signaling in a BTBR T + tf/J Mouse Model of Autism. *Mol Neurobiol* **2017**, *54*, 5201-5212, doi:10.1007/s12035-016-0066-1.

4. Krug, N.; Hohlfeld, J.M.; Kirsten, A.M.; Kornmann, O.; Beeh, K.M.; Kappeler, D.; Korn, S.; Ignatenko, S.; Timmer, W.; Rogon, C.; et al. Allergen-induced asthmatic responses modified by a GATA3-specific DNAzyme. *N Engl J Med* **2015**, *372*, 1987-1995, doi:10.1056/NEJMoa1411776.

5. Kobeissy, F.; Mallah, K.; Zibara, K.; Dakroub, F.; Dalloul, Z.; Nasser, M.; Nasrallah, L.; Mallah, Z.; El-Achkar, G.A.; Ramadan, N.; et al. The effect of clopidogrel and aspirin on the severity of traumatic brain injury in a rat model. *Neurochemistry international* **2022**, *154*, 105301, doi:10.1016/j.neuint.2022.105301.

6. Voigt, N.; Stein, J.; Galindo, M.M.; Dunkel, A.; Raguse, J.D.; Meyerhof, W.; Hofmann, T.; Behrens, M. The role of lipolysis in human orosensory fat perception. *J Lipid Res* **2014**, *55*, 870-882, doi:10.1194/jlr.M046029.

7. Andersen, D.; Storz, T.; Liu, P.; Wang, X.; Li, L.; Fan, P.; Chen, X.; Allgeier, A.; Burgos, A.; Tedrow, J.; et al. Stereoselective synthesis of a MCHr1 antagonist. *J Org Chem* **2007**, *72*, 9648-9655, doi:10.1021/jo701894v.

8. Lim, G.; You, K.Y.; Lee, J.H.; Jeon, M.K.; Lee, B.H.; Ryu, J.Y.; Oh, K.S. Identification and New Indication of Melanin-Concentrating Hormone Receptor 1 (MCHR1) Antagonist Derived from Machine Learning and Transcriptome-Based Drug Repositioning Approaches. *Int J Mol Sci* **2022**, *23*, doi:10.3390/ijms23073807.

9. Kang, J.B.; Son, H.K.; Shah, M.A.; Koh, P.O. Retinoic acid attenuates ischemic injury-induced activation of glial cells and inflammatory factors in a rat stroke model. *PloS one* **2024**, *19*, e0300072, doi:10.1371/journal.pone.0300072.

10. Viedma-Poyatos, Á.; González-Jiménez, P.; Pajares, M.A.; Pérez-Sala, D. Alexander disease GFAP R239C mutant shows increased susceptibility to lipoxidation and elicits mitochondrial dysfunction and oxidative stress. *Redox biology* **2022**, *55*, 102415, doi:10.1016/j.redox.2022.102415.

11. Yang, F.; Jing, J.J.; Fu, S.Y.; Su, X.Z.; Zhong, Y.L.; Chen, D.S.; Wu, X.Z.; Zou, Y.Q. Spinal MCP-1 Contributes to Central Post-stroke Pain by Inducing Central Sensitization in Rats. *Mol Neurobiol* **2023**, *60*, 2086-2098, doi:10.1007/s12035-022-03184-9.

12. Huang, L.; Gao, P.; Xiao, P.; Chen, Z.; Zhang, S. Transcription Factor MYB Upregulates IQGAP3 to Mediate DNA Repair and Promote 5-FU Resistance in Gastric Cancer Cells. *Drug Dev Res* **2025**, *86*, e70134, doi:10.1002/ddr.70134.

13. Ren, Y.; Liu, D.; Zhao, W.; Wang, X.; Cao, X.; Wan, W. TcMYB73, a salicylic acid-responsive R2R3-MYB transcription factor, positively regulates paclitaxel biosynthesis in Taxus chinensis in direct and indirect ways. *BMC Plant Biol* **2025**, *25*, 723, doi:10.1186/s12870-025-06755-9.

14. Shi, X.; Xu, J.; Zhong, X.; Qian, Y.; Lin, L.; Fang, Z.; Ye, B.; Lyu, Y.; Zhang, R.; Zheng, Z.; et al. Deubiquitinase MYSM1 promotes doxorubicin-induced cardiotoxicity by mediating TRIM21-ferroptosis axis in cardiomyocytes. *Cell Commun Signal* **2024**, *22*, 593, doi:10.1186/s12964-024-01955-6.

15. Lam, J.; Cazzaniga, S.; Seyed Jafari, S.M.; Maul, J.T.; Feldmeyer, L.; Bossart, S.; Yawalkar, N.; Heidemeyer, K. Treatment of Psoriasis with II-17 Inhibitors: Comparison of Long-Term Effectiveness and Drug Survival of Secukinumab vs Ixekizumab in Real-World Practice. *Psoriasis (Auckl)* **2025**, *15*, 71-84, doi:10.2147/ptt.S509495.

16. Saran, A.; Nishizaki, D.; Lippman, S.M.; Kato, S.; Kurzrock, R. Interleukin-17: A pleiotropic cytokine implicated in inflammatory, infectious, and malignant disorders. *Cytokine Growth Factor Rev* **2025**, doi:10.1016/j.cytogfr.2025.01.002.

17. Fratton, Z.; Bighetti, S.; Bettolini, L.; Maione, V.; Arisi, M.; Buligan, C.; Stinco, G.; Errichetti, E. Real-World Experience of Bimekizumab in a Cohort of 109 Patients Over 48 Weeks and Identification of Predictive Factors for an Early Super Response and Risk of Adverse Events. *Psoriasis (Auckl)* **2025**, *15*, 145-158, doi:10.2147/ptt.S514249.

18. Song, J.; Zhang, H.; Tong, Y.; Wang, Y.; Xiang, Q.; Dai, H.; Weng, C.; Wang, L.; Fan, J.; Shuai, Y.; et al. Molecular mechanism of interleukin-17A regulating airway epithelial cell ferroptosis based on allergic asthma airway inflammation. *Redox biology* **2023**, *68*, 102970, doi:10.1016/j.redox.2023.102970.

19. Reich, K.; Papp, K.A.; Blauvelt, A.; Langley, R.G.; Armstrong, A.; Warren, R.B.; Gordon, K.B.; Merola, J.F.; Okubo, Y.; Madden, C.; et al. Bimekizumab versus ustekinumab for the treatment of moderate to severe plaque psoriasis (BE VIVID): efficacy and safety from a 52-week, multicentre, double-blind, active comparator and placebo controlled phase 3 trial. *Lancet* **2021**, *397*, 487-498, doi:10.1016/s0140-6736(21)00125-2.
