# Supplementary figures and images for: Network Toxicology and Transcriptomic Analyses Reveal Ferroptosis-Related Neurotoxicity of Rotenone as an Environmental Hazardous Compound
Source: Cells. 2026 May 22;15(11):959. doi: 10.3390/cells15110959 (PMC13256928; doi:10.3390/cells15110959)

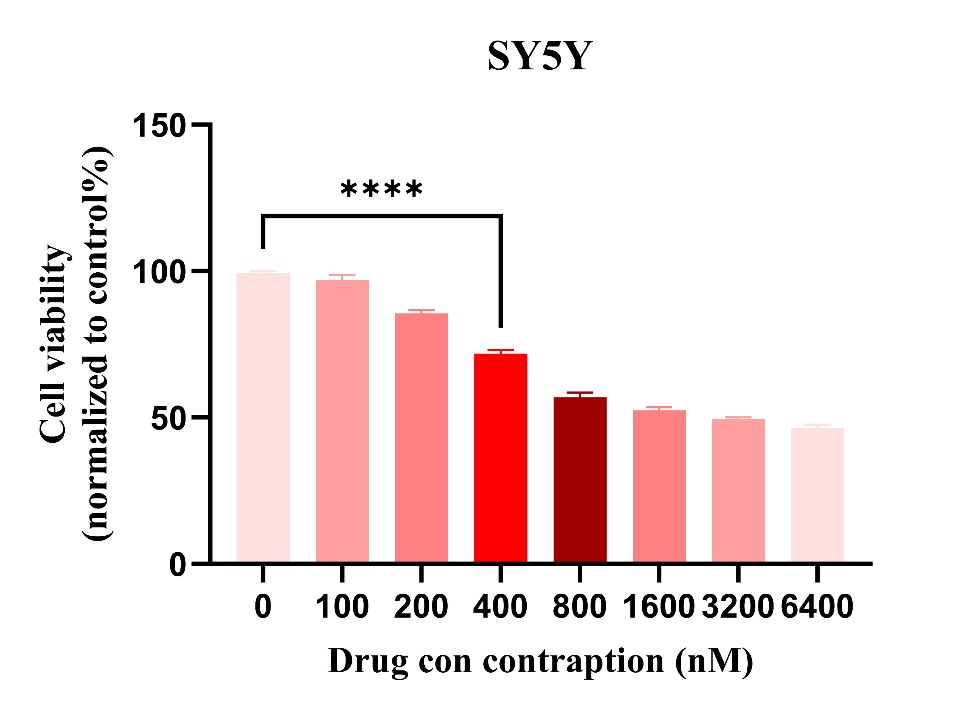

Supplement: Supplementary file 1 [file cells-15-00959-s001.zip › Supplementary Figure S1.tif]

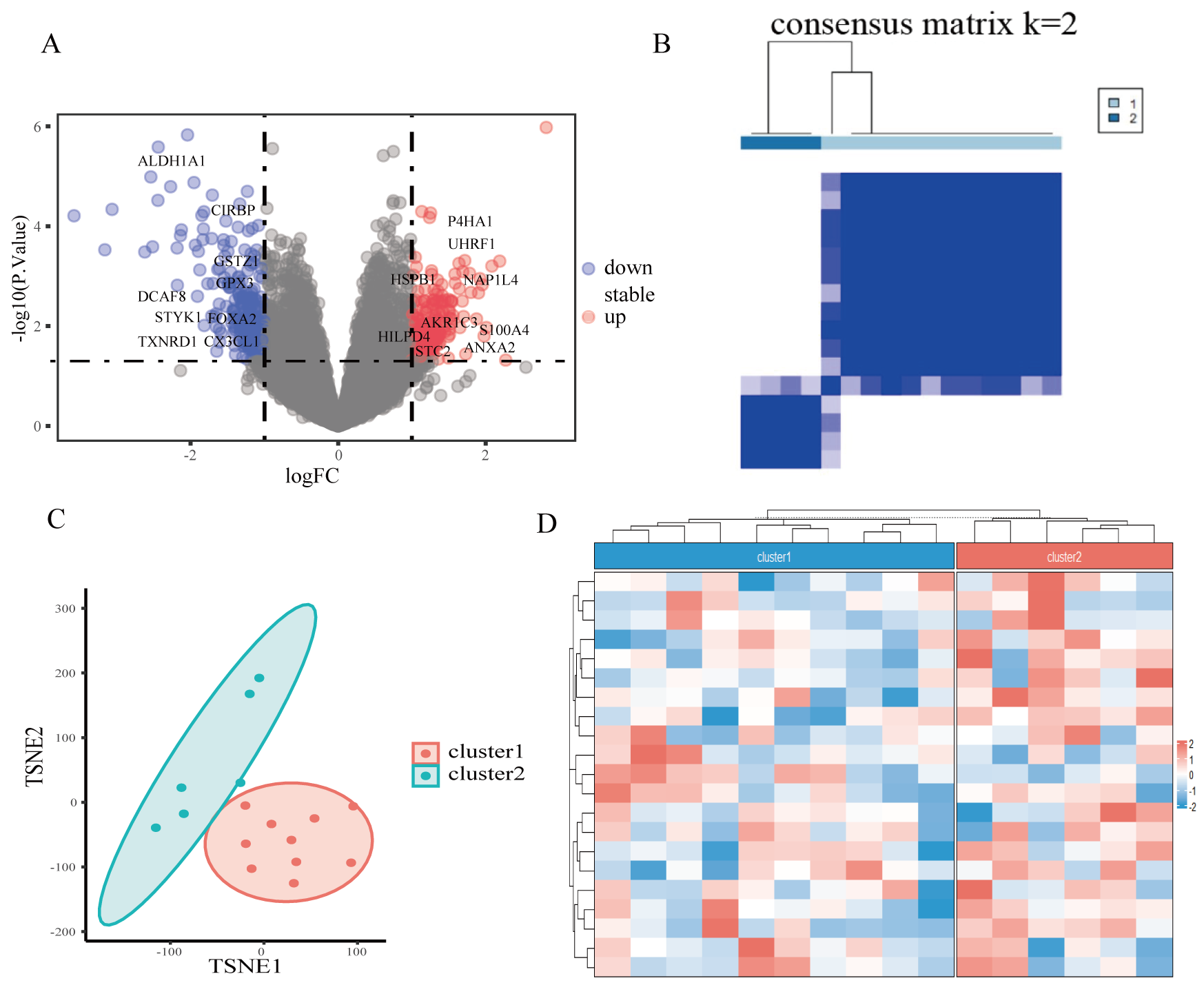

Supplement: Supplementary file 1 [file cells-15-00959-s001.zip › Supplementary Figure S2.tif]

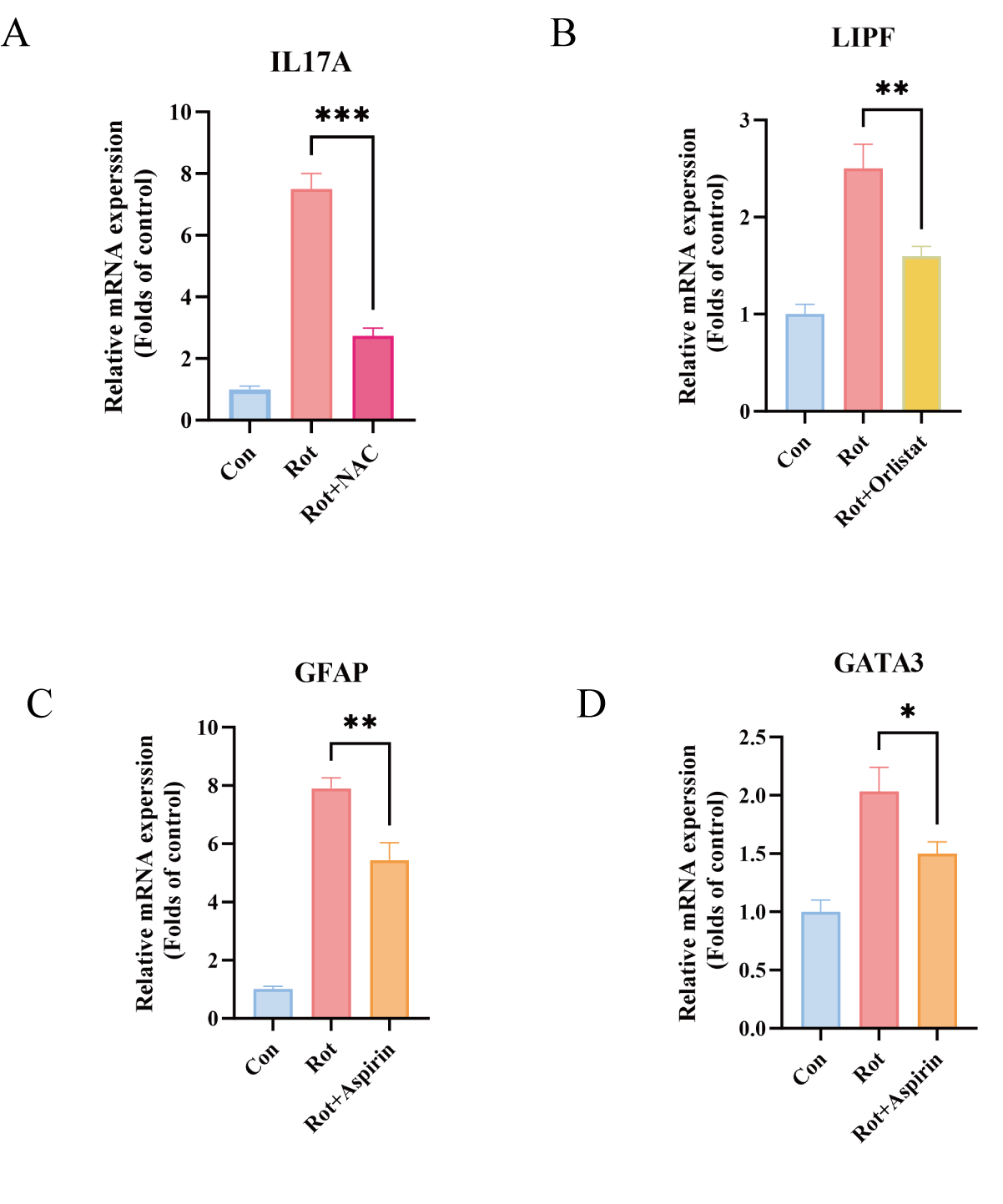

Supplement: Supplementary file 1 [file cells-15-00959-s001.zip › Supplementary Figure S3.tif]
